# Twist-related protein 1 induces epithelial-mesenchymal transition and renal fibrosis through the upregulation of complement 3

**DOI:** 10.1371/journal.pone.0272917

**Published:** 2022-08-26

**Authors:** Tomoyasu Otsuki, Noboru Fukuda, Lan Chen, Akiko Tsunemi, Masanori Abe

**Affiliations:** 1 Division of Nephrology, Hypertension and Endocrinology, Department of Medicine, Nihon University School of Medicine, Tokyo, Japan; 2 Division of Cell Regeneration and Transplantation, Department of Functional Morphology, Tokyo, Japan; University of Colorado Boulder, UNITED STATES

## Abstract

We have demonstrated that complement 3 (C3) is upregulated and induces epithelial-mesenchymal transition (EMT) phenomenon and renal fibrosis in unilateral ureteral obstruction (UUO) kidney. We investigated roles of twist-related protein 1 (TWIST1) in EMT phenomenon and renal fibrosis through C3 upregulation in a mouse UUO model with gene silencer pyrrole-imidazole (PI) polyamides targeting TWIST1. We designed and synthesized PI polyamides targeting TWIST1 binding site on mouse pre-pro C3 promoter. Increased expression C3 mRNA with interferon-γ was significantly inhibited with PI polyamide in nephrotubular epithelial cells. Immunofluorescence showed suppression of E-cadherin and enhancement of α-smooth muscle actin (α-SMA) stainings as EMT phenomena in UUO kidney. TWIST1 and C3 expression was significantly increased in UUO kidney versus contralateral unobstructed kidney (CUK). Expression of transforming growth factor-β1 (TGF-β1), α-SMA and renin mRNAs was increased in UUO kidney versus CUK. Systemic administration of TWIST1 PI polyamide significantly suppressed increased C3 expression in UUO kidney versus CUK. PI polyamide administration also suppressed the increased expression of TGF-β1, α-SMA and renin mRNAs and histologically improved renal fibrosis in UUO kidney. These findings indicate that TWIST1 induces EMT phenomenon and renal fibrosis by TGF-β1 upregulation of C3 in mouse UUO model and that TWIST1 PI polyamide may be a novel medicine for renal fibrosis.

## Introduction

Renal fibrosis is the main pathogenesis in kidney of patients with chronic kidney disease including glomerulonephritis, hypertensive nephrosclerosis and diabetic nephropathy. Epithelial-mesenchymal transition (EMT), which is characterized by a reduction in the epithelial phenotype and transition to the mesenchymal phenotype, is a pivotal phenomenon in the induction of renal fibrosis [1, 2]. Transforming growth factor-β1 (TGF-β1) is a pivotal factor that induces EMT through a reduction of E-cadherin and increase in mesenchymal markers in the nephrotubulus [3]. In addition, overexpression of complement 3 (C3) has recently been reported to reduce the expression of E-cadherin that induces the EMT phenomenon, whereas C3 knockdown increases the expression of E-cadherin in cancer tissues [4]. We previously showed that C3a induces EMT of nephrotubular epithelial cells causing a change to mesenchymal cells and increases the expression of α-SMA with increases in Krüppel-like factor 5 (KLF5) and TGF-β1 in vitro [5]. In these in vivo experiments, we showed interstitial fibrosis, tubular atrophy, and cast formation by EMT of the nephrotubulus with increases in C3 and TGF-β1 in unilateral ureteral obstruction (UUO) kidney in wild-type mice, whereas C3-deficient mice showed no renal injury, indicating that C3 induces EMT and dedifferentiation of epithelial cells to mesenchymal cells [5].

Twist-related protein 1 (TWIST1) is a transcription factor essential for the development of mesoderma and neural crest-derived craniofacial structures [6]. Recently, TWIST1 has been recognized as an important regulator of the EMT phenomenon and metastasis in cancer [7]. Cho et al. [4] reported that TWIST1 stimulates C3 and KLF-5 expressions that induce the EMT phenomenon and metastasis of ovarian cancer.

Moreover, we have also shown that C3 is increasingly expressed in mesenchymal tissues from spontaneously hypertensive rats (SHR) [8, 9]. We recently found that the expression of TWIST1 is enhanced in SHR-derived renal mesangial cells (MCs) and that the increased TWIST1 is involved in the upregulation of C3 in SHR. It is possible that TWIST1 is also involved in the EMT phenomenon to induce renal fibrosis [10].

Pyrrole-imidazole (PI) polyamides as middle molecular weight peptide compounds can form specific hydrogen bonds to double-stranded DNA (dsDNA) [11]. We previously reported that PI polyamide injected intravenously was distributed into the kidney and aorta [12]. Thus, synthetic PI polyamides designed on promoters of a target should be effective practical medicines as gene silencers. We have further shown that PI polyamides targeting TGF-β1 effectively improved renal sclerosis with hypertension [13], diabetic nephropathy in rats [14], and progressive renal diseases in the primate common marmoset [15].

In the present study, we investigated the contributions of TWIST1 to the upregulation of C3 in the EMT phenomenon and renal fibrosis in a mouse UUO model with PI polyamides targeting TWIST1.

## Materials and methods

### Designing and synthesizing of TWIST1 PI polyamides targeting C3 promoter

**Fig 1:**
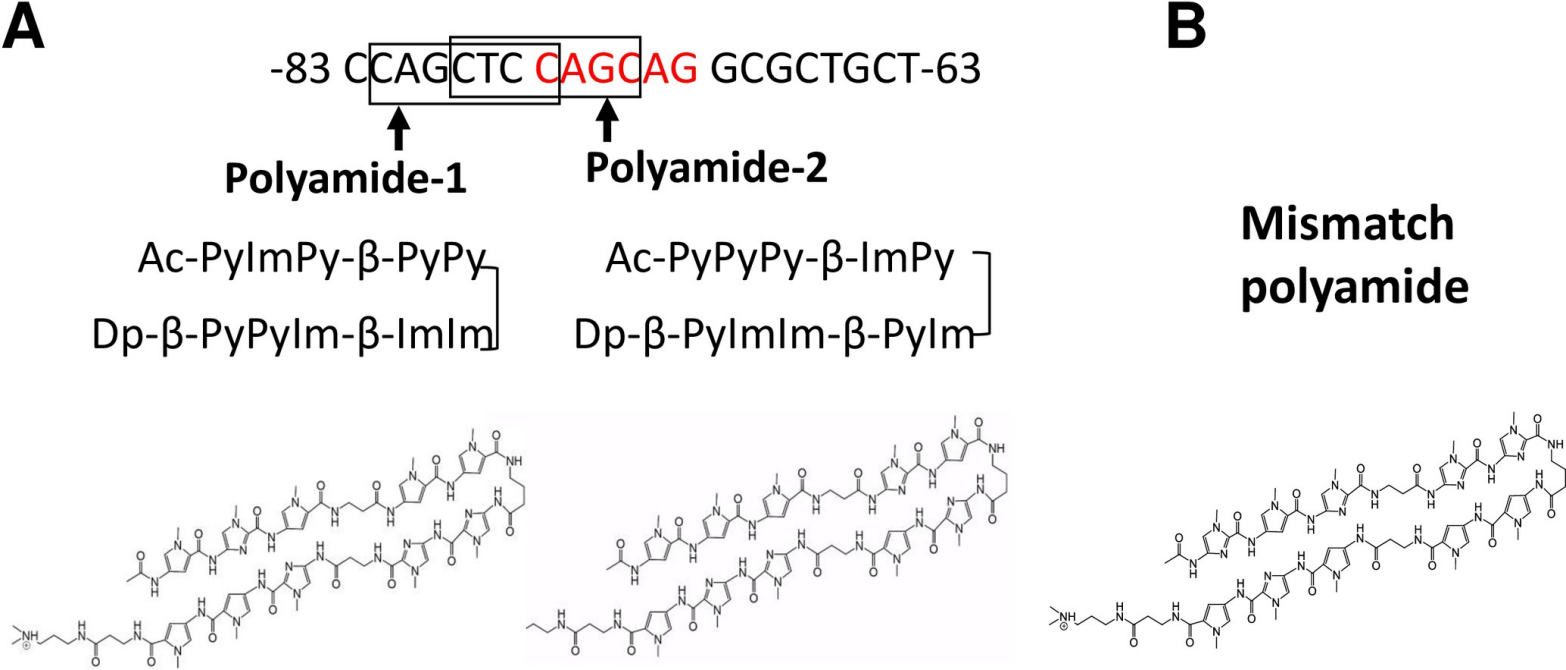
Structure of pyrrole-imidazole (PI) polyamides to prevent binding of Twist-related protein 1 (TWIST1) on the complement 3 (C3) promoter. Polyamides were synthesized by solid-phase methods and were purified by HPLC (0.1% AcOH/CH3CN 0 to 50% linear gradient, 0 to 40 minutes, 254 nm through a Chemcobond 5-ODS-H column). (A) Red capitals are TWIST1 binding sequences. Boxes indicate the binding site of PI polyamides on the C3 promoter. (B) Structure of mismatch polyamide. Py: pyrrole, Im: imidazole, Ac: acetylamide, Dp: dimethylaminopropylamide, β: β-alanine.

TWIST1 PI polyamide-1 and -2 were designed to bind across the boundary of the TWIST1 binding site on the mouse C3 promoter (-63 to -83) as reported previously [4]. Mismatch polyamide was designed not to bind transcription binding sites of the promoter (Fig 1). PI polyamides were synthesized according to previously described methods [16] and were purified by high-performance liquid chromatography.

### Gel shift and DNA binding assays for TWIST1 PI polyamides

To confirm that the designed and synthesized PI polyamides can bind target dsDNA, we performed gel shift assays. We synthesized sense oligonucleotide FITC-TCGTCGCGGACGACCTCGACC and antisense oligonucleotide GGTCGAGGTCGTCCGCGACGA. The two oligonucleotides (0.1 μM) were denatured by heating at 95°C for 5 minutes, followed by slow cooling so they could anneal with each other. The double-strand oligonucleotides and the same concentration of PI polyamide dissolved in dimethyl sulfoxide were incubated in binding buffer (20% glycerol, 5 mM MgCl2, 2.5 mM EDTA, 250 mM NaCl, 50 mM Tris-HCl [pH 7.5]) for 2 hours. The resulting complexes were separated by 20% polyacrylamide gel electrophoresis and visualized by luminescent image analyzer LAS-3000 (Fujifilm, Tokyo, Japan).

### TCMK-1 cell culture and effects of TWIST1 PI polyamides on C3 expression

TCMK-1 cells were cultured with DMEM (Sigma-Aldrich, Tokyo, Japan) including 10% FBS (Thermo Fisher Scientific). TCMK1 cells were used between passages 5 and 10th. TCMK-1 cells were stimulated with 10 ng/ml IFN-γ (R&D Systems, Minneapolis, MN, USA), and incubated with 10^−10^–10^−8^ M TWIST1 PI polyamide-1 or PI polyamide-2 dissolved in dimethyl sulfoxide and incubated for 3 hours.

### Real time PCR analysis

In the in vitro experiments, total RNA was extracted from cultured TCMK-1 cells by ISOGEN (Nippon Gene, Toyama, Japan), whereas in the in vivo experiments, total RNA was extracted from mouse renal medulla by TRIzol^TM^ Reagent (Invitrogen, Waltham, MA, USA). Aliquots of total RNA were reverse transcribed into single-stranded cDNA by incubation with avian myeloblastoma virus reverse transcriptase (Takara Biochemicals, Osaka, Japan). Diluted cDNA products were then subjected to PCR. A real-time PCR analysis was performed by the TaqMan probe method with TaqMan Primer of Rn00585470_s1 for Twist1 mRNA, Mm01232779_m1 for C3 mRNA, Mm01178820_m1 for Tgfβ1, or Mm00725412_s1 for Acta2 (α-SMA) mRNA (Applied Biosystems). Primers and probes of Ren1c (renin) were (sense, 5′-CCCTCCCCGACATTTCCT-3′; antisense, 5′-GCACGTAGTCCGTACTGCTGAG-3′; and probe, 5′-TGACCTGGGAGGCAGGGCCTACATAMRA-3′). An ABI Prism 7300 (Applied Biosystems) was used to quantify mRNA. Each sample (each reaction, 5 μl complementary DNA; total volume, 25 μl) was run in triplicate. To control sample loading, we determined mouse 18S ribosomal RNA levels with TaqMan Ribosomal RNA Control Reagents (Applied Biosystems). Amplifications were done at 95°C for 20 seconds, 60°C for 1 second, and 72°C for 120 seconds with a GenAmp PCR System 2700 (Applied Biosystems) for 35 cycles. After we determined the threshold cycle (Ct), we used the comparative Ct method to calculate the relative quantification of mRNA expression of the marker gene. The quality and concentration of amplified PCR products were determined using an Agilent 2100 Bioanalyzer (Agilent, Palo Alto, CA, USA).

### Ethics and animals

This investigation conformed to the Guide for the Care and Use of Laboratory Animals published by the US National Institutes of Health (NIH Publication No. 85–23, 1996). The ethics committee of the Nihon University School of Medicine approved the research protocols involving the use of living animals in this study (no. AP17M035-1), which was conducted in accordance with the Guidelines for Conducting Animal Experiments of the CIEA. Male C57BL/6J mice were obtained from Japan Charles River Laboratories (Yokohama, Kanagawa, Japan).

### Creation of UUO and in vivo experimental protocol

The 8-week-old male C57BL/6J mice were anesthetized with 50 mg/kg body weight ketamine and 100 mg/kg body weight xylazine. The left ureter was exposed via a lateral incision and ligated by two sutures at the level of the lower renal pole.

The experimental protocol is shown in S1 Fig. Control mice (n = 6) received 0.01% acetic acid through the tail vein twice a week for two weeks. Polyamide mice (n = 8) or Mismatch mice (n = 8) received 1 mg/kg of TWIST1 PI polyamide or Mismatch PI polyamide dissolved in 0.01% acetic acid through the tail vein twice a week for two weeks. Systolic blood pressure was measured in prewarmed unanesthetized mice by tail-cuff plethysmography (Natsume Seisakusho, Tokyo, Japan) before creation of UUO, at 5, 10, and 14 days after the creation of UUO. Mice were killed at 14 days after the operation. Both CUK and UUO kidney were collected and assessed for morphology, immunohistochemistry, and gene expression.

### Histopathological and immunohistochemical examinations

Tissue samples from the kidney were fixed in 10% neutral buffered formalin solution and then embedded in paraffin. Then, 4-μm-thick sections were stained with hematoxylin-eosin or Masson trichrome (MT). MT-positive area was calculated from a sum of the representative MT-stained areas using Photoshop CS3 extended (Adobe Systems Inc., San Jose, CA, USA). The sections were subsequently deparaffinized and dehydrated using a routine procedure. For immunohistochemical analysis, the primary antibodies used were follows: mouse monoclonal anti-E-cadherin (BD Bioscience, Franklin Lake, NJ, USA) diluted 1:100, rabbit polyclonal anti-α-SMA (Abcam, Cambridge, UK) diluted 1:400, rabbit polyclonal antibody against TWIST1 (Abcam) diluted 1:500, goat anti-mouse complement C3 (Thomas Scientific, Swedesboro, NJ, USA) diluted 1:1000, and mouse monoclonal anti-TGF-β1, 2, 3 (R&D Systems) diluted 1:100. Then, Alexa Fluor 594 anti-goat secondary antibody (Thermo Fisher Scientific, Waltham, MA, USA) diluted 1:200 or Qdot 655 F(ab’)2-Goat anti-Rabbit IgG (Thermo Fisher Scientific) diluted 1:100 for E-cadherin or α-SMA was used as secondary antibodies. For TWIST1, C3, and TGF-β1 staining, sections were incubated with peroxidase-labeled mouse and rabbit polyclonal goat antibody (Sigma-Aldrich). Counterstaining was then performed before the sections were examined under a light microscope.

### Western blot analysis

Renal medulla from mice were disrupted with lysis buffer (50 mM Tris·HCl, pH 8.0, 150 mM NaCl, 0.02% sodium azide, 100 μg/ml phenylmethylsulfonyl fluoride, 1 μg/ml aprotinin, and 1% Triton X-100). Total proteins were extracted and purified with 100 μl of chloroform and 400 μl of methanol. Protein samples were boiled for 3 minutes and subjected to electrophoresis on 8% polyacrylamide gels and then transblotted to nitrocellulose membranes (Bio-Rad Laboratories). Blots were incubated with anti-TWIST1 antibody (Abcam) diluted 1:1000. C3 antibody (Santa Cruz Biotechnology, Santa Cruz, CA, USA) diluted 1:100, anti-TGF-β1 antibody (Abcam) diluted 1:500, anti-renin antibody diluted 1:200 (Santa Cruz), anti-α-SMA antibody (Abcam) diluted 1:500, anti-E-cadherin antibody diluted 1:500 (Calbiochem Biosciences, La Jolla, CA, USA) and anti-beta actin antibody (Abcam) diluted 1:40,000 in 5% non-fat milk in TBST solution (10 mM Tris·HCl, pH 8.0, 150 mM NaCl, and 0.05% Tween 20) for 3 hours at room temperature. The membrane was incubated with horseradish peroxidase-labeled secondary antibody (Sigma-Aldrich, St. Louis, MO, USA) for 1 hours at room temperature and then washed with TBST once for 15 minutes and then four more times for 5 minutes each. Immune complexes on the membrane were detected by the enhanced chemiluminescence method (Amersham Pharmacia Biotech, Little Chalfont, Buckinghamshire, UK). Each protein was quantified and normalized by β-actin using the Image J software program (National Institutes of Health, Bethesda, MD, USA).

### Statistical analysis

Values are reported as means ± standard deviation (SD). Statistical differences within groups or between two groups were assessed by Student *t*-test using SPSS version 24 software (SPSS Inc., Chicago, IL, USA). *P* values < .05 were considered to indicate statistical significance.

## Results

### Gel mobility shift assay for TWIST1 PI polyamides

**Fig 2:**
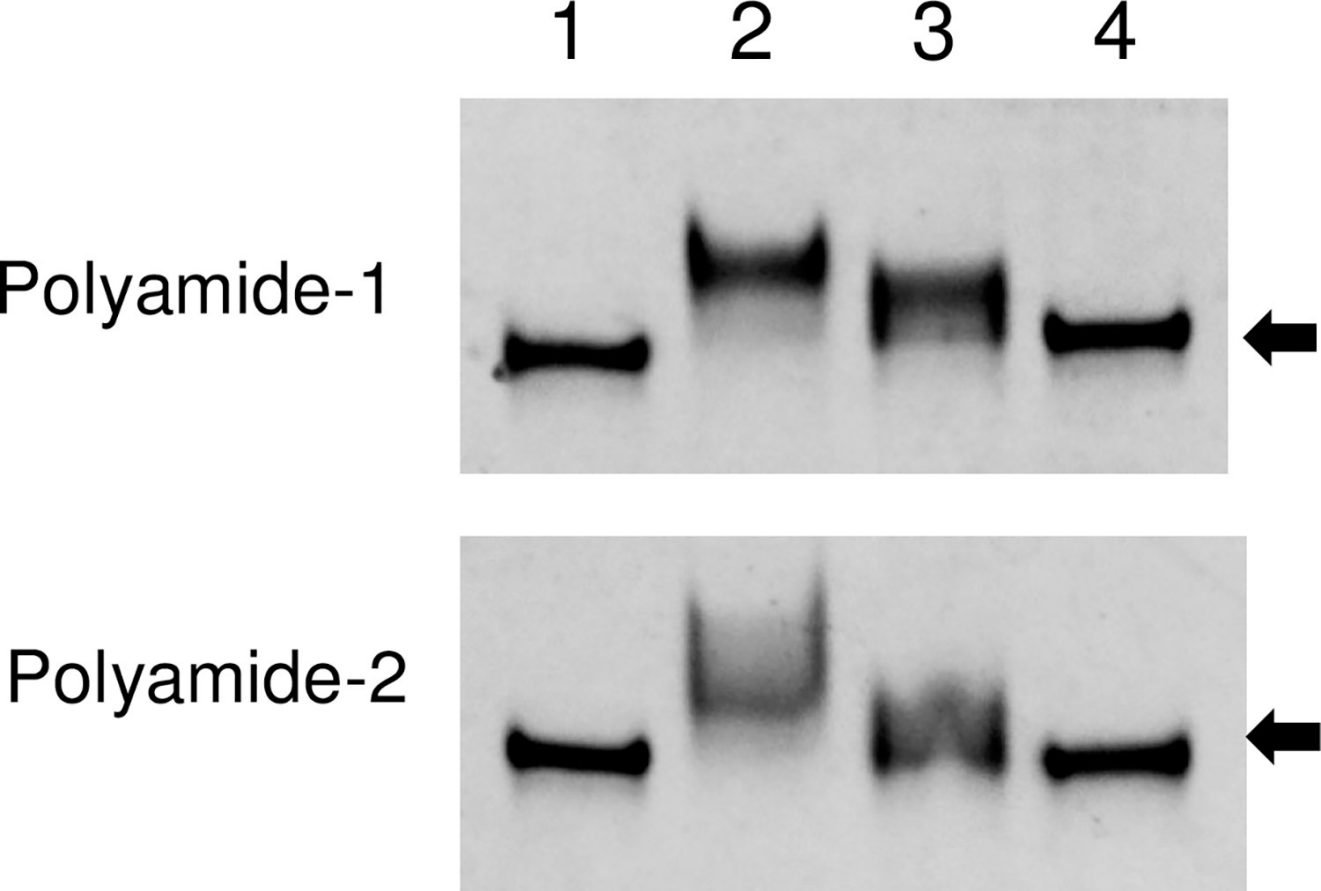
Gel mobility shift and DNA binding assays for Twist-related protein 1 (TWIST1) pyrrole-imidazole (PI) polyamides. The 21-mer sense oligonucleotide and antisense oligonucleotide for TWIST1 PI polyamides (Polyamide-1 and -2). The double-strand oligonucleotides and the same concentration of PI polyamide dissolved in dimethyl sulfoxide were incubated in binding buffer. Lane 1: FITC-dsDNA (10–5 M), Lane 2: FITC-dsDNA (10–5 M) + TWIST1 PI polyamide (10–5 M), Lane 3: FITC-dsDNA (10–5 M) + excessive dsDNA (10–3 M) + TWIST1 PI polyamide (10–5 M), Lane 4: FITC-dsDNA (10–5 M) + mismatch PI polyamide (10–5 M). Arrows indicate the basal position of the dsDNA.

Bindings of the two PI polyamides designed to span the boundary of TWIST1 binding sequences of the C3 promoter on target dsDNAs were evaluated by gel mobility shift assay. TWIST1 PI polyamide-1 and -2 bound the appropriate 21-bp FITC-labeled dsDNAs, which are indicated by the shift of bands (Lane 2) shown in Fig 2. The addition of excessive non-FITC labeled dsDNAs showed both shifted and non-shifted bands (Lane 3). TWIST1 PI polyamide-1 and -2 did not bind mismatch dsDNAs (Lane 4). These findings indicated that the two synthesized PI polyamides can bind target dsDNA.

### Effects of TWIST1 PI polyamides on IFN-γ-stimulated expression of C3 mRNA

**Fig 3:**
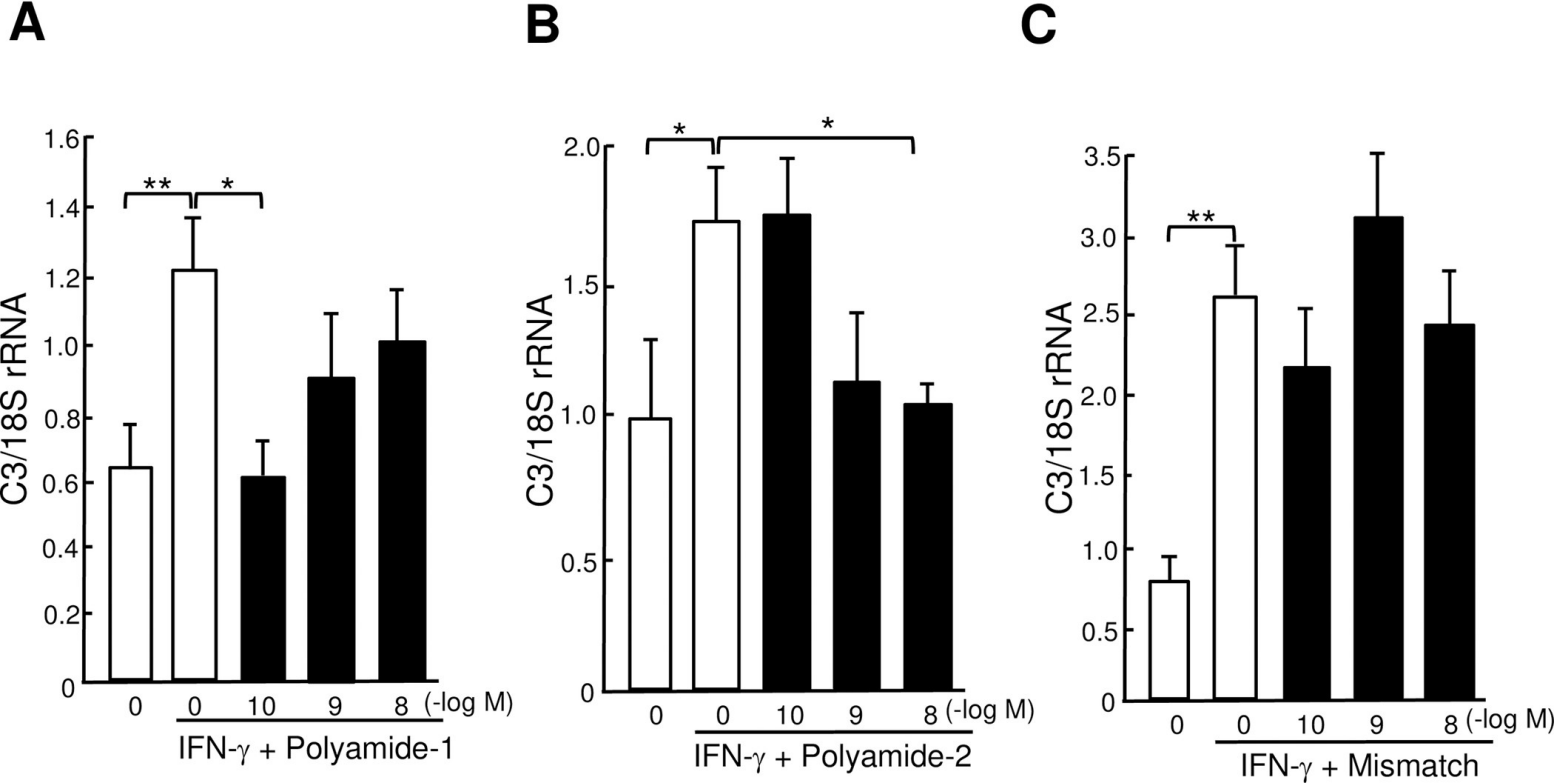
Effects of Twist-related protein 1 (TWIST1) pyrrole-imidazole (PI) polyamides on interferon (IFN)-γ-stimulated expression of complement 3 (C3) mRNA. TCMK-1 cells were stimulated with 10 ng/ml IFN-γ and incubated with 10^−10^–10^−8^ M TWIST1 (A) PI polyamide-1, (B) PI polyamide-2 or (C) Mismatch PI polyamide and incubated for 3 hours. Data are the mean ± SD (n = 4). * *P* < .05, ** *P* < .01 between indicated columns.

Interferon (IFN)-γ (10 ng/ml) significantly (*P* < .05) increased abundabces of C3 mRNA in TCMK-1 mouse nephrotubular epithelial cells. Concentrations of 10^−10^ M of PI polyamide-1 and 10^−8^ M PI polyamide-2 significantly (*P* < .05) decreased IFN-γ-stimulated abundances of C3 mRNA in cells (Fig 3A and 3B). Mismatch polyamide did not affect IFN-γ-stimulated abundances of C3 mRNA (Fig 3C). We used PI polyamide-1 to suppress TWIST1 gene expression in the following in vivo experiments.

### Changes in blood pressure and body weight in UUO mice with TWIST1 PI polyamide

Systolic blood pressure was significantly (*P* < .05) elevated in mice after the creation of UUO. Administration of TWIST1 PI polyamide did not affect the elevation of blood pressure in the UUO mice. However, after the creation of UUO, body weight in the UUO mice increased with TWIST1 PI polyamide (S2 Fig).

### Immunofluorescence of E-cadherin and α-SMA in UUO kidney

Staining of E-cadherin was positive in the nephrotubulus of contralateral unobstructed kidney (CUK), whereas E-cadherin was not stained in the nephrotubulus of UUO kidney. Staining of α-SMA was negative in CUK but was enhanced in renal medulla in UUO kidney, indicating the occurrence of the EMT phenomenon in renal medulla of UUO kidney (S3 Fig).

### Effects of TWIST1 PI polyamide on expression of Twist1, C3 and Tgf-β1 mRNAs in UUO kidney

**Fig 4:**
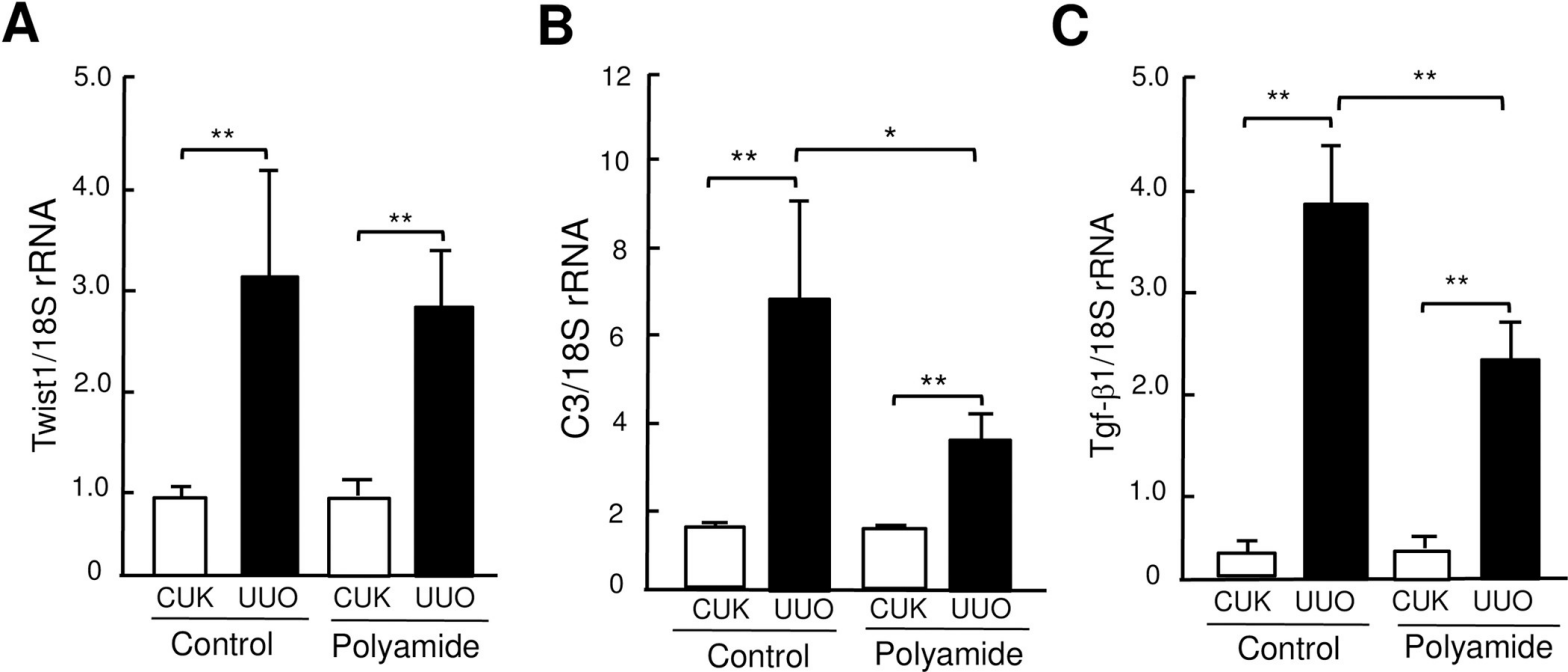
Real-time PCR analysis for expression of (A) twist-related protein 1 (Twist1), (B) complement 3 (C3) and (C) transforming growth factor-β1 (Tgf-β1) mRNAs in renal medulla of contralateral unobstructed kidney (CUK) and unilateral ureteral obstruction (UUO) kidney without or with TWIST1 pyrrole-imidazole (PI) polyamide. Control mice (n = 6) received 0.01% acetic acid via tail vein twice a week for two weeks. Polyamide mice (n = 8) received 1 mg/kg of TWIST1 PI polyamide via tail vein twice a week for two weeks. Data are the mean ± SD. * *P* < .05, ** *P* < .01 between indicated columns.

The abundance of Twist1 mRNA was significantly (*P* < .01) higher in UUO kidney than that in CUK. TWIST1 PI polyamide did not affect the increased Twist1 mRNA in UUO kidney (Fig 4A). The abundance of C3 mRNA was significantly (*P* < .01) higher in UUO kidney than that in CUK. Administration of TWIST1 PI polyamide significantly (*P* < .05) decreased the increased abundance of C3 mRNA in UUO kidney (Fig 4B). The abundance of Tgf-β1 mRNA was significantly (*P* < .01) higher in UUO kidney than that in CUK. Administration of TWIST1 PI polyamide significantly (*P* < .01) inhibited the increased abundance of Tgf-β1 mRNA in UUO kidney (Fig 4C).

### Effects of TWIST1 PI polyamide on expression of TWIST1, C3 and TGF-β1 proteins in UUO kidney

**Fig 5:**
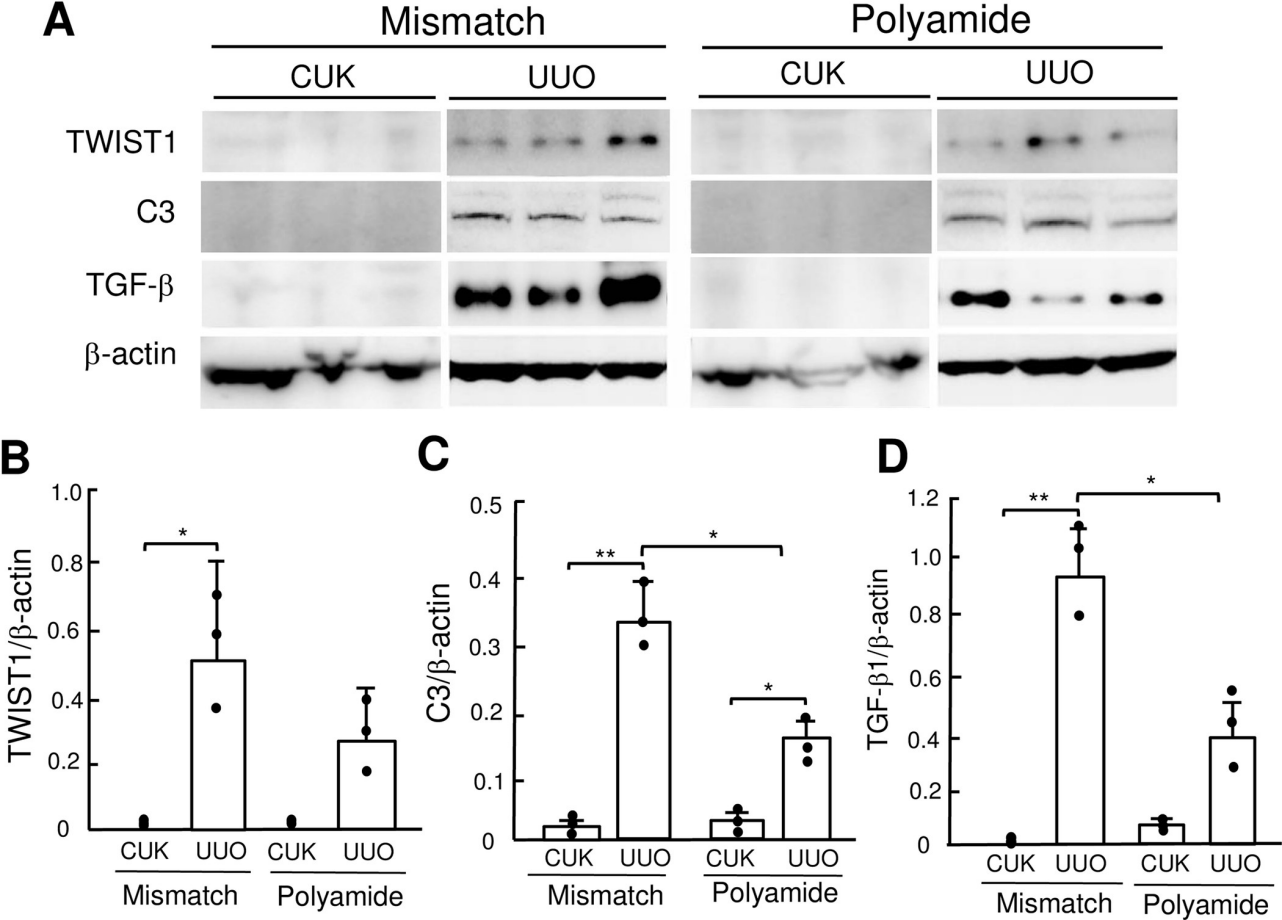
Western blot analysis for expression of (A, B) Twist-related protein 1 (TWIST1), (A, C) complement 3 (C3) and (A, D) transforming growth factor-β1 (TGF-β1) proteins in renal medulla of contralateral unobstructed kidney (CUK) and unilateral ureteral obstruction (UUO) kidney without or with TWIST1 pyrrole-imidazole (PI) polyamide. Control mice received 0.01% acetic acid via tail vein twice a week for two weeks. Polyamide or Mismatch mice received 1 mg/kg of TWIST1 PI polyamide or Mismatch PI polyamide via tail vein twice a week for two weeks. Each protein was quantified and normalized by β-actin using the Image J software program. Data are the mean ± SD (n = 3). * *P* < .05, ** *P* < .01 between indicated columns.

The abundance of TWIST1 protein was significantly (*P* < .05) higher in UUO kidney than that in CUK. TWIST1 PI polyamide decreased, but not statistically significantly, the amount of increased TWIST1 protein in UUO kidney (Fig 5A and 5B). The abundance of C3 protein was significantly (*P* < .01) higher in UUO kidney than that in CUK. Administration of TWIST1 PI polyamide significantly (*P* < .05) decreased the increased amount of C3 protein in UUO kidney (Fig 5A and 5C). The abundance of TGF-β1 protein was significantly (*P* < .01) higher in UUO kidney than that in CUK. Administration of TWIST1 PI polyamide significantly (*P* < .05) decreased the increased amount of TGF-β1 protein in UUO kidney (Fig 5A and 5D).

### Effects of TWIST1 PI polyamide on TWIST1, C3 and TGF-β1 immunostainings in UUO kidney

**Fig 6:**
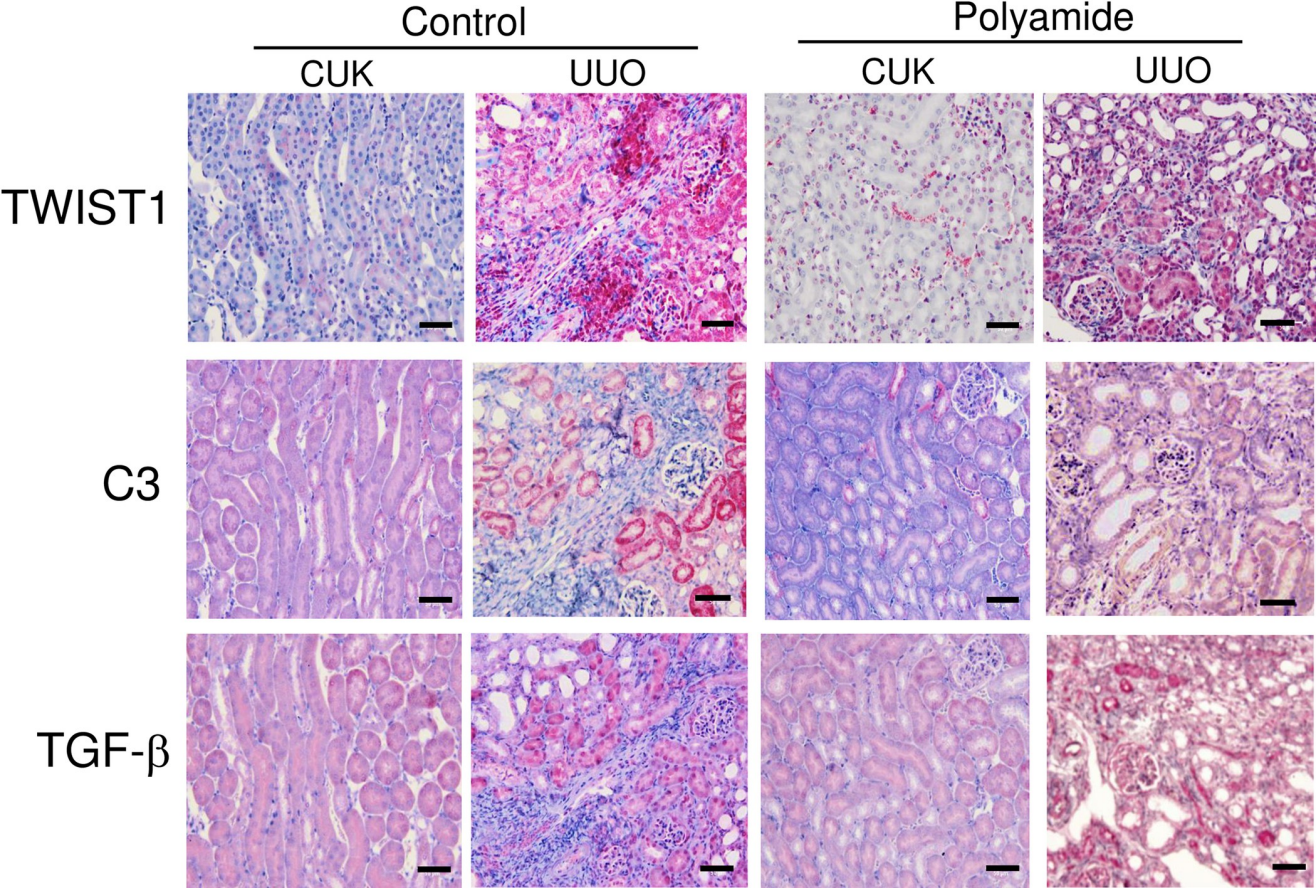
Immunohistochemical staining of Twist-related protein 1 (TWIST1), complement 3 (C3) and transforming growth factor-β1 (TGF-β1) (red color) on contralateral unobstructed kidney (CUK) and unilateral ureteral obstruction (UUO) kidney without or with TWIST1 pyrrole-imidazole (PI) polyamide. Control mice received 0.01% acetic acid via tail vein twice a week for two weeks. Polyamide mice received 1 mg/kg of TWIST1 PI polyamide via tail vein twice a week for two weeks. Represents are typical images from four samples. Scale bar = 50 μm.

Immunohistochemistry showed that staining of TWIST1 was stronger on nephrotubulus in UUO kidney than that in CUK. Staining of C3 was stronger on nephrotubulus in UUO kidney than that in CUK. TWIST1 PI polyamide suppressed the increase in C3 staining in UUO kidney. Staining of TGF-β1 was stronger on nephrotubulus in UUO kidney than that in CUK. TWIST1 PI polyamide did not appreciably affect the increase in TGF-β1 staining in UUO kidney (Fig 6).

### Effects of TWIST1 PI polyamide on renal fibrosis in UUO kidney

**Fig 7:**
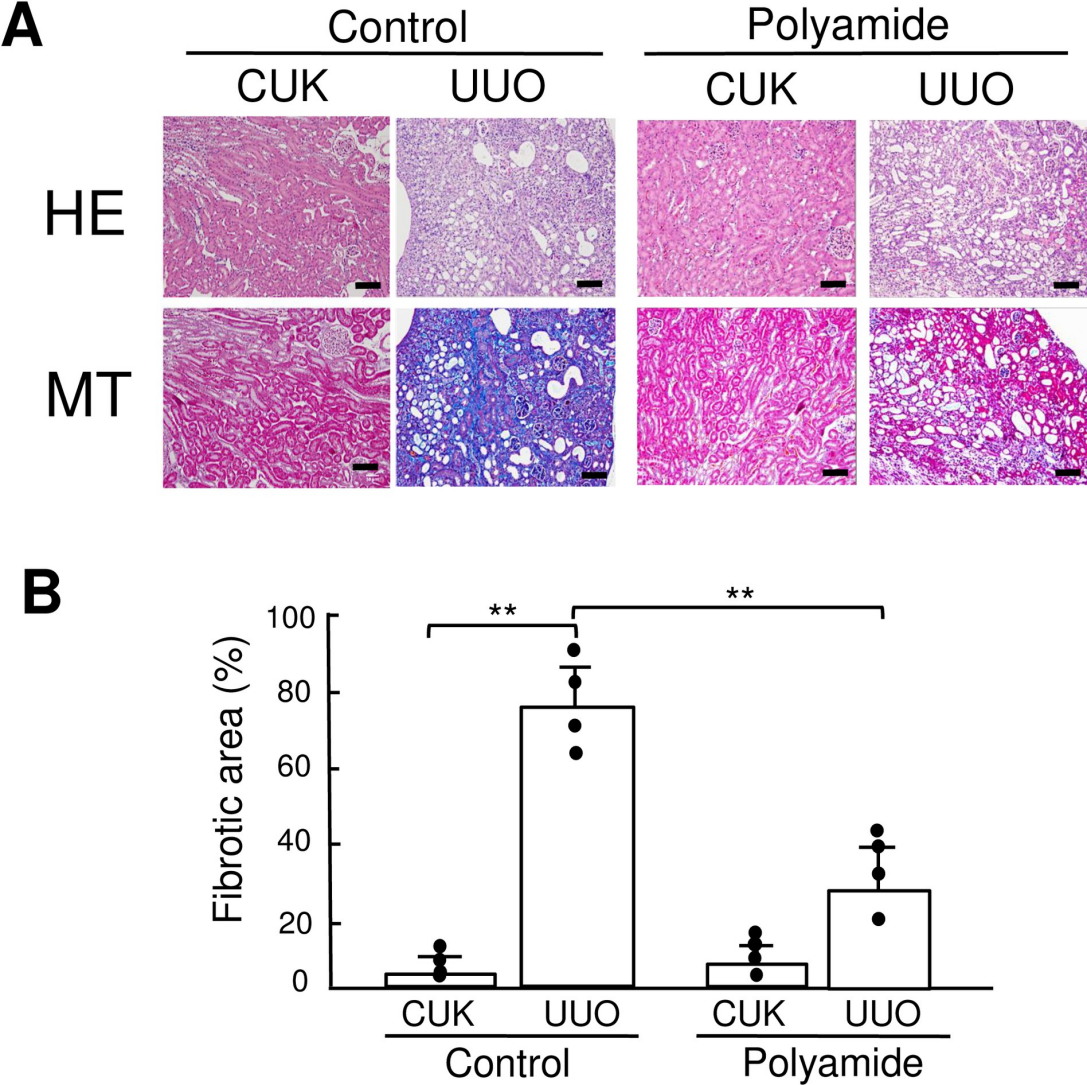
Effects of Twist-related protein 1 (TWIST1) pyrrole-imidazole (PI) polyamide (Polyamide) on renal fibrosis in unilateral ureteral obstruction (UUO) kidney. Control mice received 0.01% acetic acid via tail vein twice a week for two weeks. Polyamide mice received 1 mg/kg of TWIST1 PI polyamide via tail vein twice a week for two weeks. (A) Tissue samples from the kidney were stained with hematoxylin-eosin (HE) or Masson trichrome (MT) in contralateral unobstructed kidney (CUK) and UUO kidney. (B) MT-positive area was calculated from a sum of the representative MT-stained areas. Data are the mean ± SD (n = 4). ** *P* < .01 between indicated columns. Scale bar = 50 μm.

Fig 7 shows HE and Masson trichrome stainings of UUO kidney and CUK from mice treated with or without TWIST1 PI polyamide. In contrast to CUK in the control mice, UUO kidney showed degeneration of renal medulla that included interstitial fibrosis, tubular atrophy, and cast formation. Administration of TWIST1 PI polyamide markedly reduced interstitial fibrosis, tubular atrophy, and cast formation in UUO kidneys compared to those in control mice (Fig 7A). Fibrotic area was significantly (*P* < .01) higher in UUO kidney than CUK. Administration of TWIST1 PI polyamide significantly (*P* < 0.01) decreased fibrotic area in UUO kidney (Fig 7B).

### Effects of TWIST1 PI polyamide on EMT phenomen and renin expression in UUO kidney

**Fig 8:**
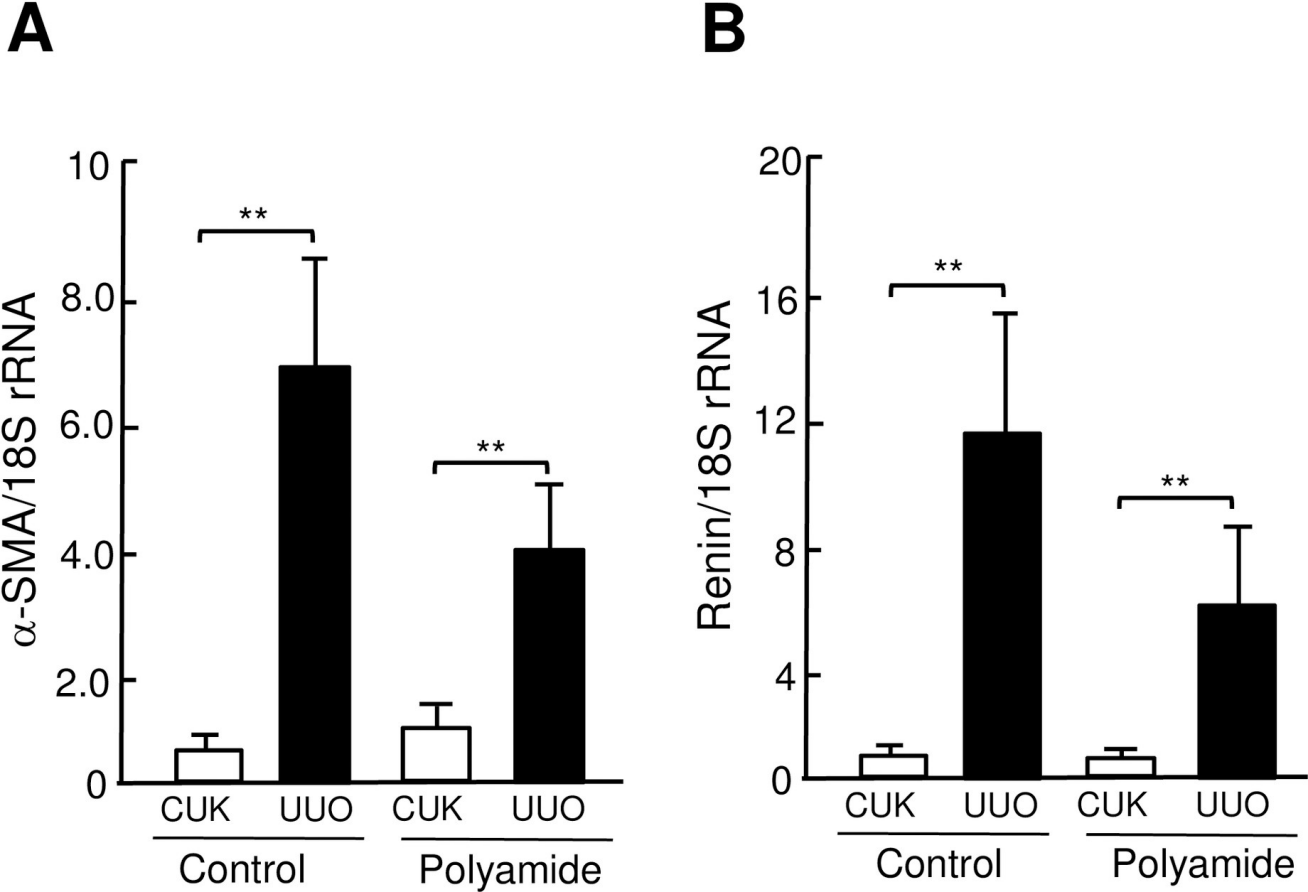
Effects of Twist-related protein 1 (TWIST1) pyrrole-imidazole polyamide (Polyamide) on expression of α-SMA (A) and renin (B) mRNA in unilateral ureteral obstruction (UUO) kidney. Control mice (n = 6) received 0.01% acetic acid via tail vein twice a week for two weeks. Polyamide mice (n = 8) received 1 mg/kg of TWIST1 pyrrole-imidazole polyamide via tail vein twice a week for two weeks. Expression of α-SMA and renin mRNAs was determined by real-time PCR analysis. Data are the mean ± SD. * *P* < .05, ** *P* < .01 between indicated columns.

**Fig 9:**
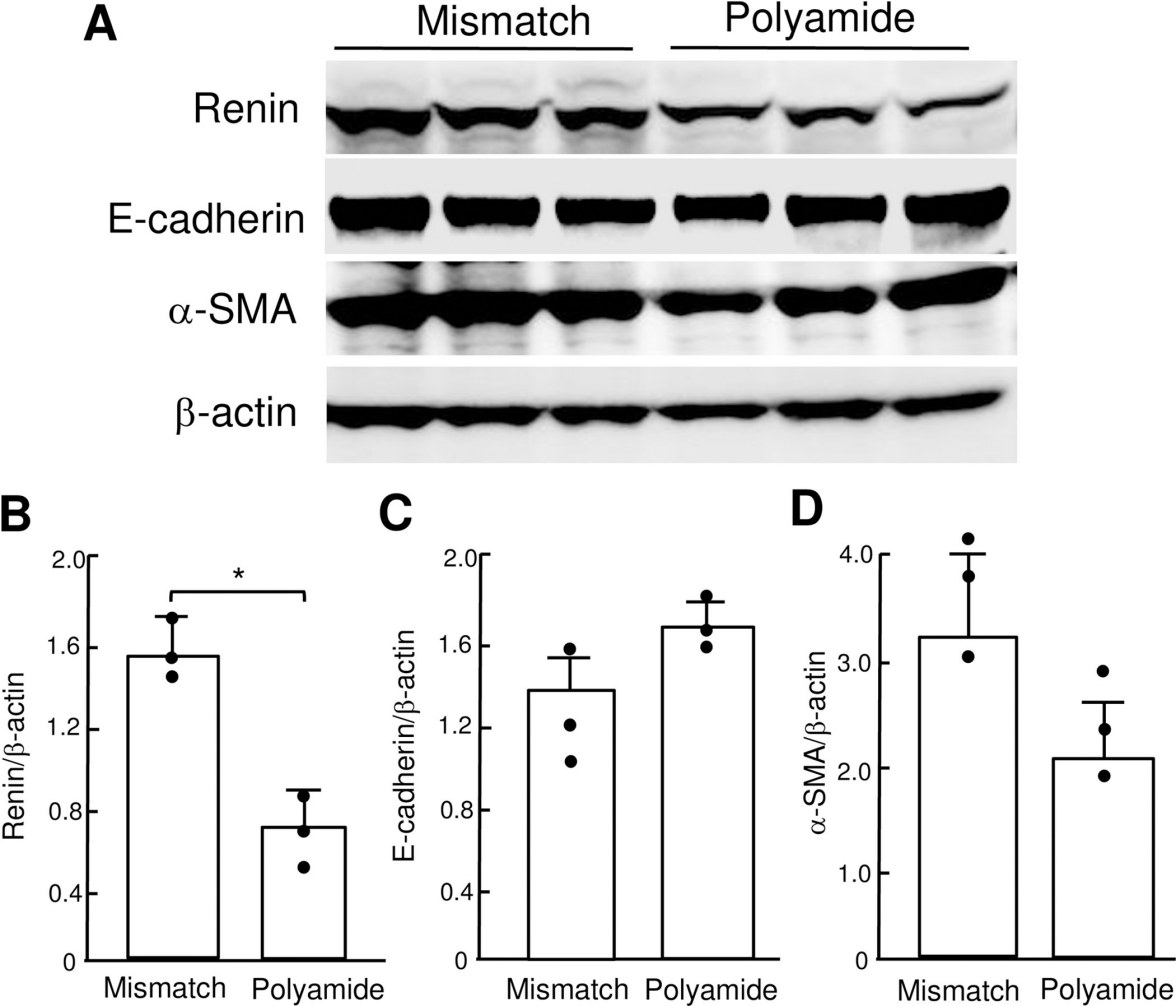
(A) Western blot analysis for expression of renin, E-cadherin and α-smooth muscle actin (α-SMA) proteins in renal medulla of unilateral ureteral obstruction (UUO) kidney without or with TWIST1 pyrrole-imidazole (PI) polyamide. Control mice received 0.01% acetic acid via tail vein twice a week for two weeks. Polyamide or Mismatch mice received 1 mg/kg of TWIST1 PI polyamide or Mismatch PI polyamide via tail vein twice a week for two weeks. (B) Each protein was quantified and normalized by β-actin using the Image J software program. Data are the mean ± SD (n = 3). * *P* < .05, between indicated columns.

Abundance of α-SMA and renin mRNAs was significantly (*P* < .01) higher in UUO kidney than that in CUK. Administration of TWIST1 PI polyamide suppressed the increase in amount of α-SMA and renin mRNAs in UUO kidney (Fig 8). Fig 9 shows effects of TWIST1 PI polyamide on amount of renin, E-cadherin and α-SMA proteins in renal medulla of UUO kidney. Administration of TWIST1 PI polyamide significantly (*P* < .05) decreases amount of renin protein in UUO kidney. Administration of TWIST1 PI polyamide did not affect amount of E-cadhelin protein in UUO kidney.

## Discussion

We recently investigated the role of TWIST1 in the increased expression of C3 in MCs from SHR. TWIST1 siRNA significantly decreased the expression of C3 mRNA in MCs from SHR, indicating that TWIST1 upregulates the C3 gene in MCs from SHR [10]. In the present experiments, to evaluate the involvement of TWIST1 as a transcription factor in upregulation of the C3 gene, we examined the effects of gene silencer PI polyamides in preventing TWIST1 binding on the expression of C3 and Twist1 mRNAs in TCMK-1 cells stimulated with IFN-γ. PI polyamides targeting TWIST1 significantly suppressed the IFN-γ-stimulated expression of C3 in TCMK-1 cells, indicating that TWIST1 transcriptionally upregulates C3 gene.

Immunofluorescence staining in the in vivo experiments showed decreases in E-cadherin staining and increases in α-SMA staining implicating the obvious EMT phenomenon in UUO kidney. In addition, expression of TWIST1 and C3 was increased with increases in the expression of TGF-β1, α-SMA and renin mRNAs in UUO kidney. It is possible that TWIST1 upregulates C3 to induce the EMT phenomenon in UUO kidney. Lovisa et al. [17] find that during renal fibrosis tubular epithelial cells acquire a partial EMT program during which they remain associated with their basement membrane and express markers of both epithelial and mesenchymal cells. Deletion of Twist1 or Snai1 in proximal tubular epithelial cells resulted in inhibition of the EMT program and the maintenance of tubular epithelial cell integrity. Thus, inhibition of EMT program in tubular epithelial cells during chronic renal injury represents a potential anti–fibrosis therapy.

To confirm the contribution of TWIST1 on the upregulation of C3 in vivo, we intravenously administered TWIST1 PI polyamide in the mouse UUO model. Administration of PI polyamide significantly decreased the expression of C3 and TGF-β1 and decreased that of α-SMA mRNA. In addition, TWIST1 PI polyamide significantly increased E-cadherin protein in UUO kidney. These results suggest that TWIST1 transcriptionally upregulates the C3 gene, which increases TGF-β1 to induce the EMT phenomenon in UUO kidney.

Moreover, the expression of renin was elevated in UUO kidney and was suppressed with the administration of TWIST1 PI polyamide. In our previous study, upregulated C3 induced the EMT phenomenon with TGF-β1, by which nephrotubular epithelial cells were transformed to mesenchymal cells that produce renin with nuclear translocation of LXRα in mouse UUO kidney. Moreover, the produced renin increased intrarenal angiotensin II and elevated the blood pressure in the mouse UUO model. In contrast, the translocation of LXRα and increases in intrarenal renin and angiotensin II were not observed in UUO kidney in the C3-deficient mice [5]. Blood pressure was elevated in mice after creation of UUO in the present experiments. These findings and present experimental results suggest that TWIST1 transcriptionally upregulates C3 to induce the EMT phenomenon in the nephrotubulus and activate the renal renin-angiotensin system to elevate blood pressure in UUO mice.

We previously showed that C3 is increasingly expressed in mesenchymal tissues from SHR [8]. We targeted the C3 gene by using gene-editing technology in SHR and found that the increased C3 induces salt-sensitive hypertension with increases in the intrarenal activation of the renin-angiotensin system by the dedifferentiation of mesenchymal tissues in kidney from SHR [18]. Thus, C3 induces the synthetic phenotype of mesenchymal cells through the activation of KLF5, and EMT of the nephrotubulus to generate renin and angiotensin II in SHR. More recently, we evaluated the formation of renin-producing cells and the roles of C3 in renin generation during differentiation of mesenchymal stem cells and found that C3 maintains an undifferentiated state of smooth muscle cells from mesenchymal stem cells to generate renin that activates the renin-angiotensin system [19]. It is thought that TWIST1 upregulates C3 to induce the EMT phenomenon that activates the intrarenal renin-angiotensin system and is associated the pathogenesis of hypertension.

In the present experiments, the administration of TWIST1 PI polyamide inhibited protein expression of TWIST1 in UUO kidney. As PI polyamides were designed to prevent binding of TWIST1 on the C3 promoter, the expression of TWIST1 is not theoretically affected by the PI polyamides. TWIST1 has been reported to be present downstream of TGF-β signalings, in which TWIST1 directly suppresses E-cadherin to induce the EMT phenomenon [20, 21]. Thus, it is possible that the administration of TWIST1 PI polyamide inhibits the expression of TGF-β1 and then suppresses the expression of TWIST1 protein in UUO kidney.

Renal fibrosis resulting from the EMT phenomenon is also induced by TGF-β and is the main pathogenesis of chronic kidney diseases. The systemic administration of TWIST1 PI polyamide considerably improved renal fibrosis in UUO kidney, indicating that the TWIST1-C3 system is involved in renal fibrosis through the EMT phenomenon.

Middle-molecular-weight peptide compounds have been expected to be the next-generated medicines because of their specificity and stability [22]. PI polyamides, as one of these peptide compounds, fully resist nuclease-induced biological degradation and do not require vector-assisted delivery systems because they can easily permeate cells and enter the nuclei. Thus, as novel DNA-recognized agents, PI polyamides may be more applicable than nucleic acid medicines [23]. Thus, it is feasible that TWIST1 PI polyamide could become a practical medicine in the treatment of human CKD.

**Fig 10:**
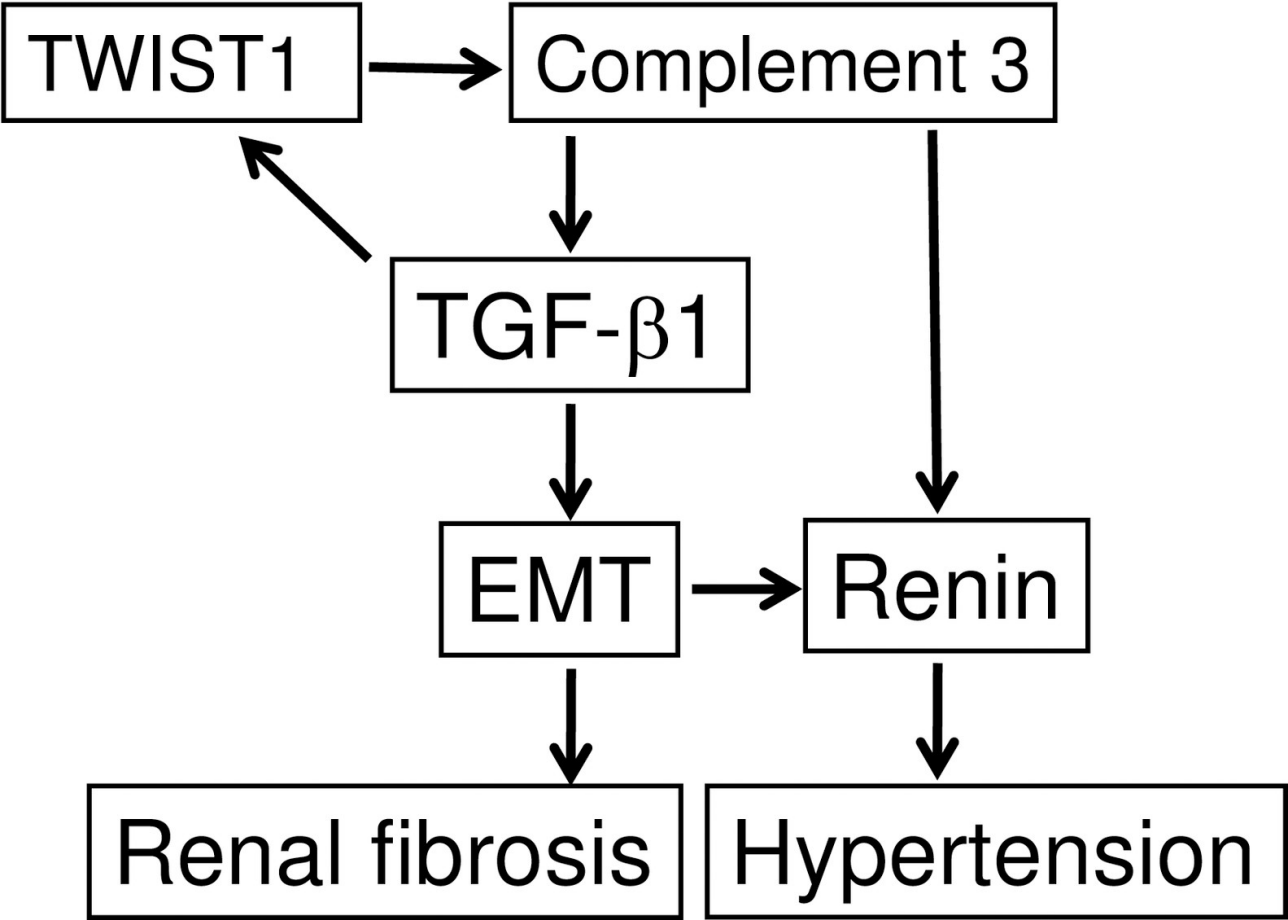
A diagram of the involvement of Twist-related protein 1 (TWIST1) in the epithelial-mesenchymal transition (EMT) phenomenon and renal fibrosis. TGF-β1: transforming growth factor-β1.

Fig 10 presents a diagram of the involvement of TWIST1 in the EMT phenomenon and renal fibrosis. TWIST1 upregulates C3 that stimulates the expression of TGF-β1, which induces the EMT phenomenon to cause renal fibrosis. TGF-β1 also stimulates the expression of TWIST1, and C3 stimulates the expression of renin to induce hypertension.

## Conclusions

TWIST1 induced the EMT phenomenon and renal fibrosis by upregulation of C3 with TGF-β1 in a mouse UUO model. TWIST1 PI polyamide may become a novel medicine for the treatment of renal fibrosis.

## Supporting information

S1 Fig Experimental protocol of the effects of Twist-related protein 1 (TWIST1) pyrrole-imidazole (PI) polyamide on unilateral ureteral obstruction (UUO) mice. Control mice (n = 6) received 0.01% acetic acid via tail vein twice a week for two weeks. Polyamide or Mismatch mice (n = 8) received 1 mg/kg of TWIST1 or Mismatch PI polyamide via tail vein twice a week for two weeks indicated by arrows. At 2 weeks after UUO creation, mice were killed to remove their kidneys.
(PDF)

S2 Fig Changes in blood pressure and body weight in unilateral ureteral obstruction (UUO) mice with Twist-related protein 1 (TWIST1) pyrrole-imidazole (PI) polyamide. Control mice (n = 8) received 0.01% acetic acid via tail vein twice a week for two weeks. Polyamide mice (n = 6) received 1 mg/kg of TWIST1 PI polyamide via tail vein twice a week for two weeks. Systolic blood pressure was elevated by tail cuff method in mice before and 5, 10, and 14 days after creation of UUO. * *P* < .05 vs day 0.
(PDF)

S3 Fig Immunofluorescence of E-cadherin and α-smooth muscle actin (SMA) in unilateral ureteral obstruction (UUO) kidney. Both CUK and UUO kidneys were collected and assessed for immunofluorescence staining with E-cadherin or α-SMA antibodies. Scale bar = 50 μm.
(PDF)

S1 Raw images (PDF)
