# Supplementary material for: Twist-related protein 1 induces epithelial-mesenchymal transition and renal fibrosis through the upregulation of complement 3
Source: PLoS One. 2022 Aug 26;17(8):e0272917. doi: 10.1371/journal.pone.0272917 (PMC9417022; doi:10.1371/journal.pone.0272917)
Supplement: S1 Fig — Control mice (n = 6) received 0.01% acetic acid via tail vein twice a week for two weeks. Polyamide or Mismatch mice (n = 8) received 1 mg/kg of TWIST1 or Mismatch PI polyamide via tail vein twice a week for two weeks indicated by arrows. At 2 weeks after UUO creation, mice were killed to remove their kidneys. (PDF) [file pone.0272917.s001.pdf]

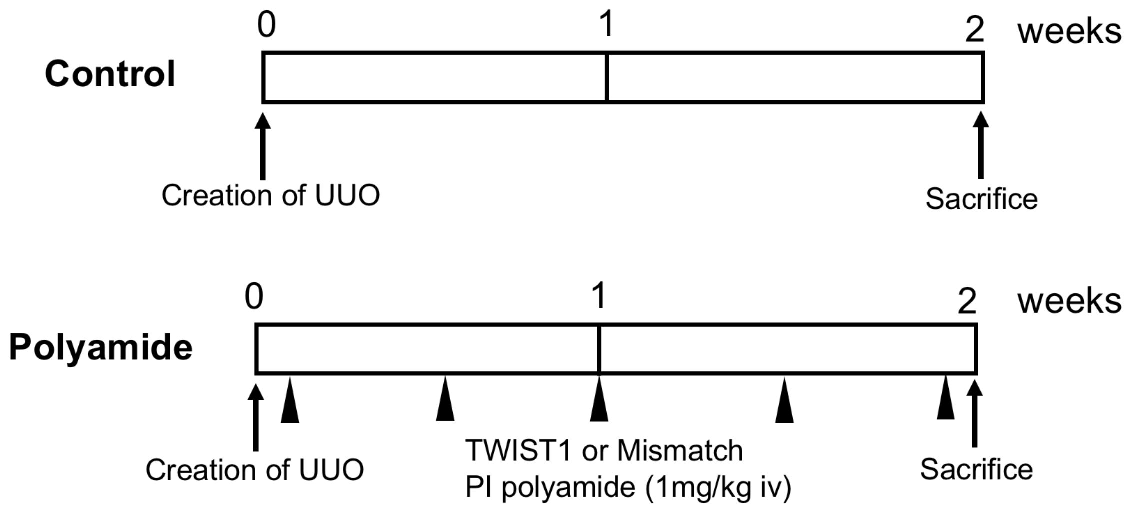

S1 Fig. Experimental protocol of the effects of Twist-related protein 1 (TWIST1) pyrrole-imidazole (PI) polyamide on unilateral ureteral obstruction (UUO) mice. Control mice (n=6) received 0.01% acetic acid via tail vein twice a week for two weeks. Polyamide or Mismatch mice (n=8) received 1 mg/kg of TWIST1 or Mismatch PI polyamide via tail vein twice a week for two weeks indicated by arrows. At 2 weeks after UUO creation, mice were killed to remove their kidneys.
