# Supplementary material for: Twist-related protein 1 induces epithelial-mesenchymal transition and renal fibrosis through the upregulation of complement 3
Source: PLoS One. 2022 Aug 26;17(8):e0272917. doi: 10.1371/journal.pone.0272917 (PMC9417022; doi:10.1371/journal.pone.0272917)
Supplement: S2 Fig — Control mice (n = 8) received 0.01% acetic acid via tail vein twice a week for two weeks. Polyamide mice (n = 6) received 1 mg/kg of TWIST1 PI polyamide via tail vein twice a week for two weeks. Systolic blood pressure was elevated by tail cuff method in mice before and 5, 10, and 14 days after creation of UUO. * P < .05 vs day 0. (PDF) [file pone.0272917.s002.pdf]

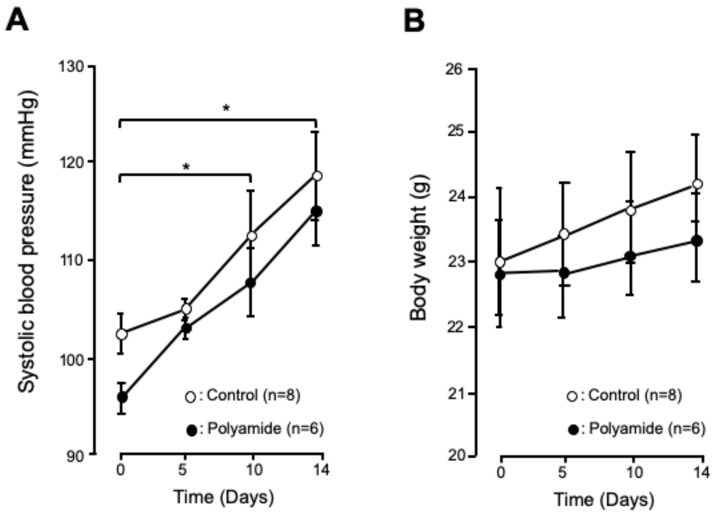

S2 Fig. Changes in blood pressure and body weight in unilateral ureteral obstruction (UUO) mice with Twist-related protein 1 (TWIST1) pyrrole-imidazole (PI) polyamide. Control mice (n=8) received 0.01% acetic acid via tail vein twice a week for two weeks. Polyamide mice (n=6) received 1 mg/kg of TWIST1 PI polyamide via tail vein twice a week for two weeks. Systolic blood pressure was elevated by tail cuff method in mice before and 5, 10, and 14 days after creation of UUO. * $P < .05$ vs day 0.
