# Supplementary material for: Twist-related protein 1 induces epithelial-mesenchymal transition and renal fibrosis through the upregulation of complement 3
Source: PLoS One. 2022 Aug 26;17(8):e0272917. doi: 10.1371/journal.pone.0272917 (PMC9417022; doi:10.1371/journal.pone.0272917)
Supplement: S3 Fig — Both CUK and UUO kidneys were collected and assessed for immunofluorescence staining with E-cadherin or α-SMA antibodies. Scale bar = 50 μm. (PDF) [file pone.0272917.s003.pdf]

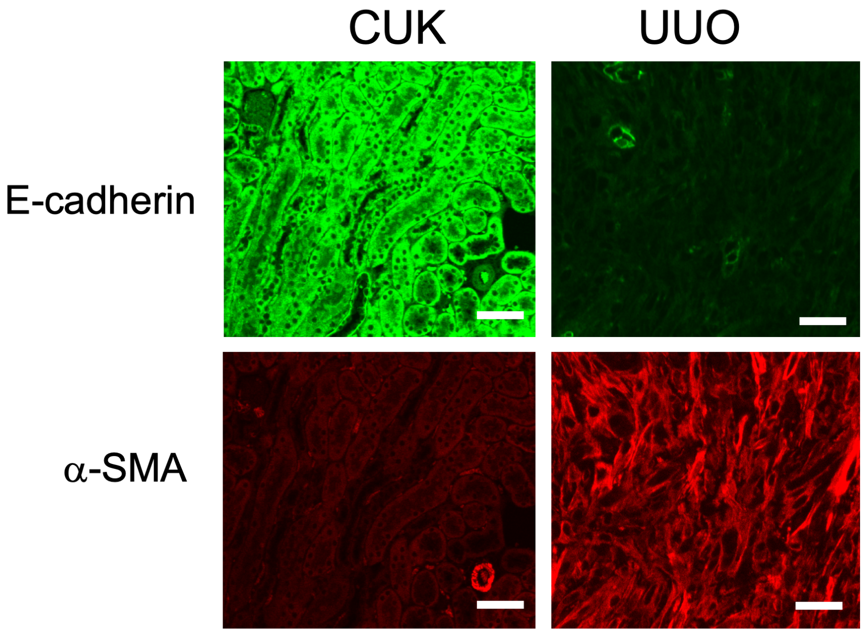

S3 Fig. Immunohistochemistry for epithelial mesenchymal transition (EMT) markers E-cadherin and α-smooth muscle actin (α-SMA) in renal medulla of contralateral unobstructed kidney (CUK) and unilateral ureteral obstruction (UUO) kidney. Both CUK and UUO kidneys were collected and assessed for immunofluorescence staining with E-cadherin or α-SMA antibodies. Scale bar = 50 μm.
