# Supplementary material for: Twist-related protein 1 induces epithelial-mesenchymal transition and renal fibrosis through the upregulation of complement 3
Source: PLoS One. 2022 Aug 26;17(8):e0272917. doi: 10.1371/journal.pone.0272917 (PMC9417022; doi:10.1371/journal.pone.0272917)

Western blot for TWIST1, C3 , TGF-β and β-actin in CUK kidney with or without PI polyamide and Mismatch polyamide

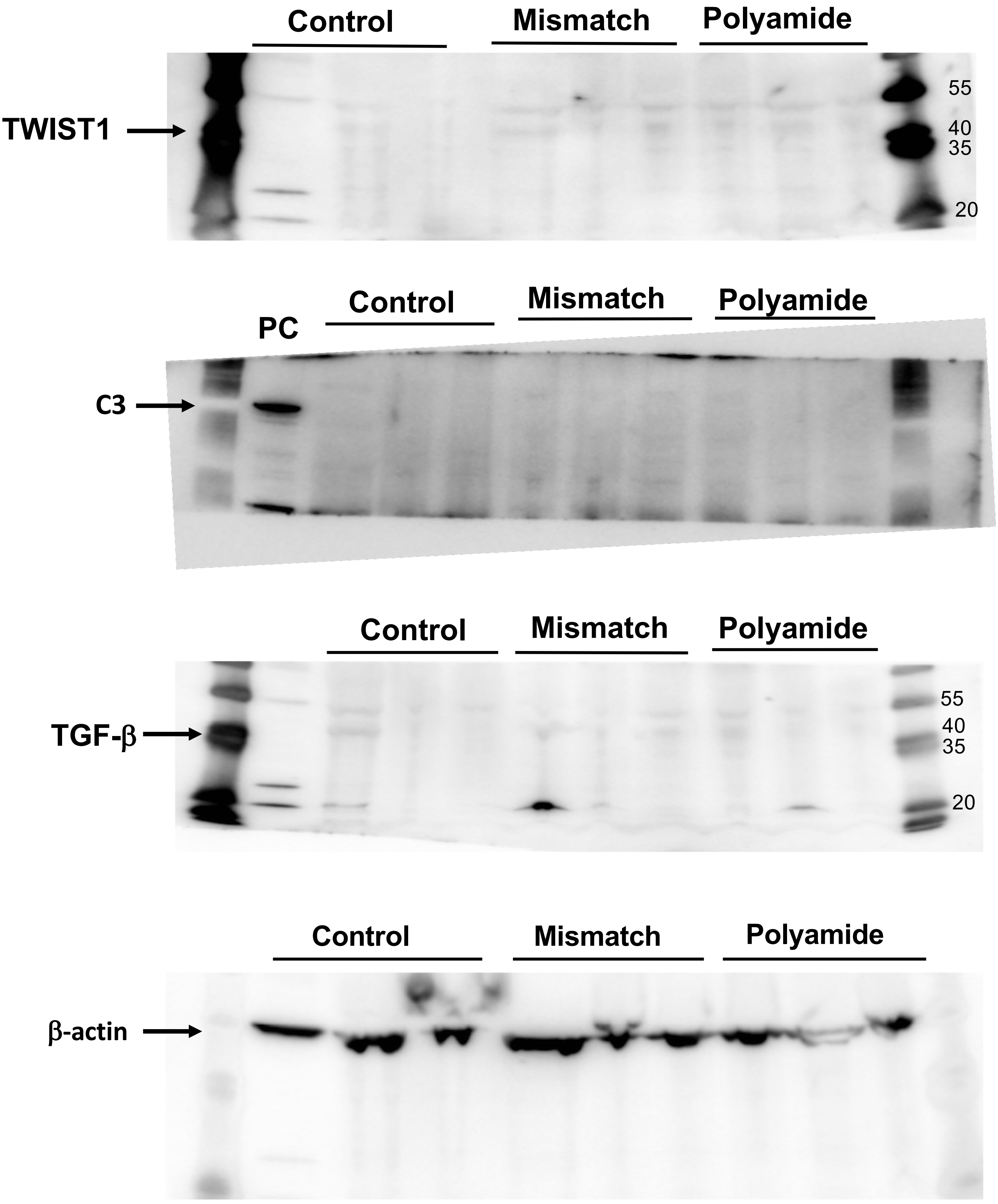

Western blot for TWIST1, C3 , TGF-β and renin in UUO kidney with or without PI polyamide and Mismatch polyamide

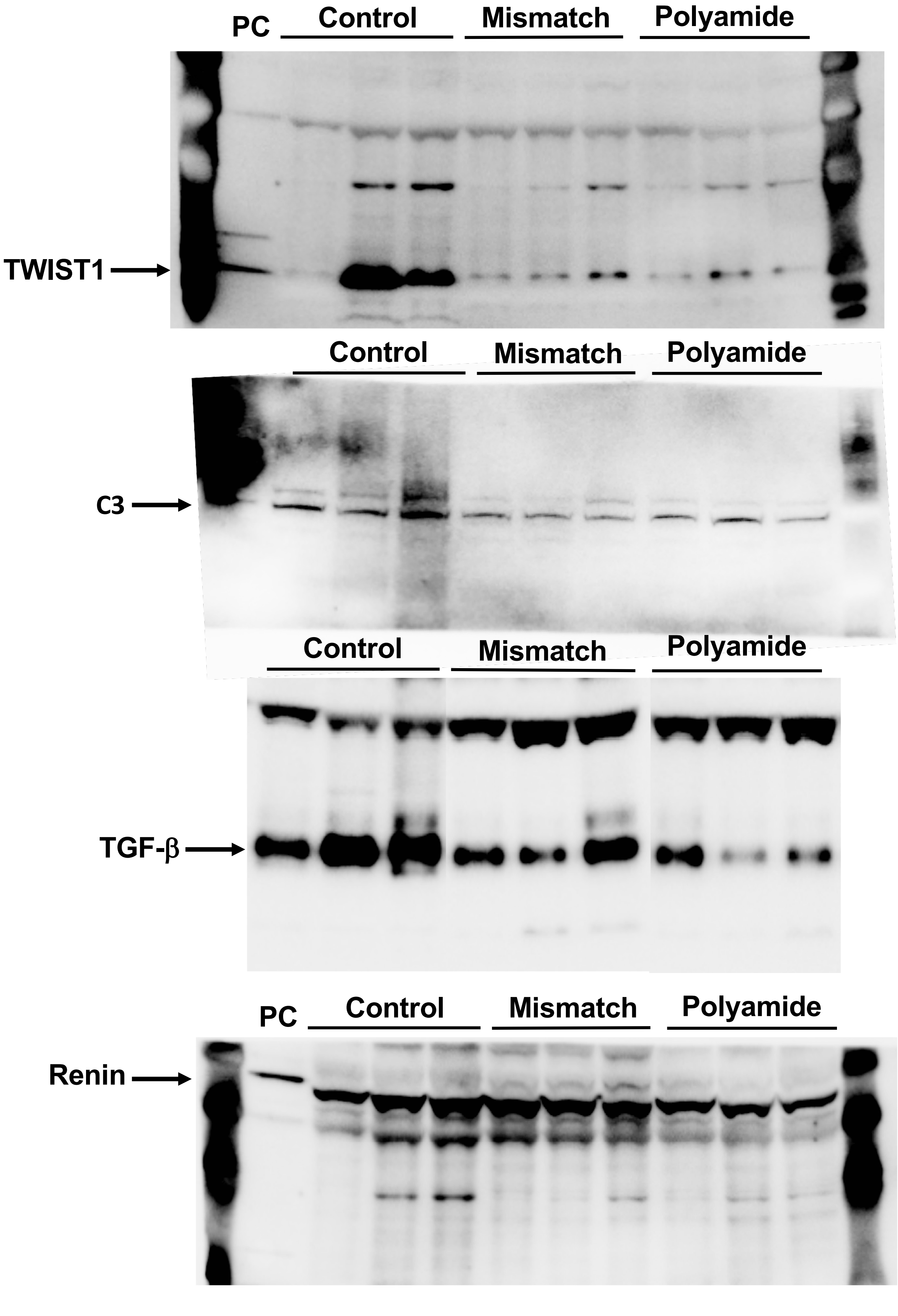

Western blot for E-cadhelin, α-SMA and β-actin in UUO kidney with or without PI polyamide and Mismatch polyamide

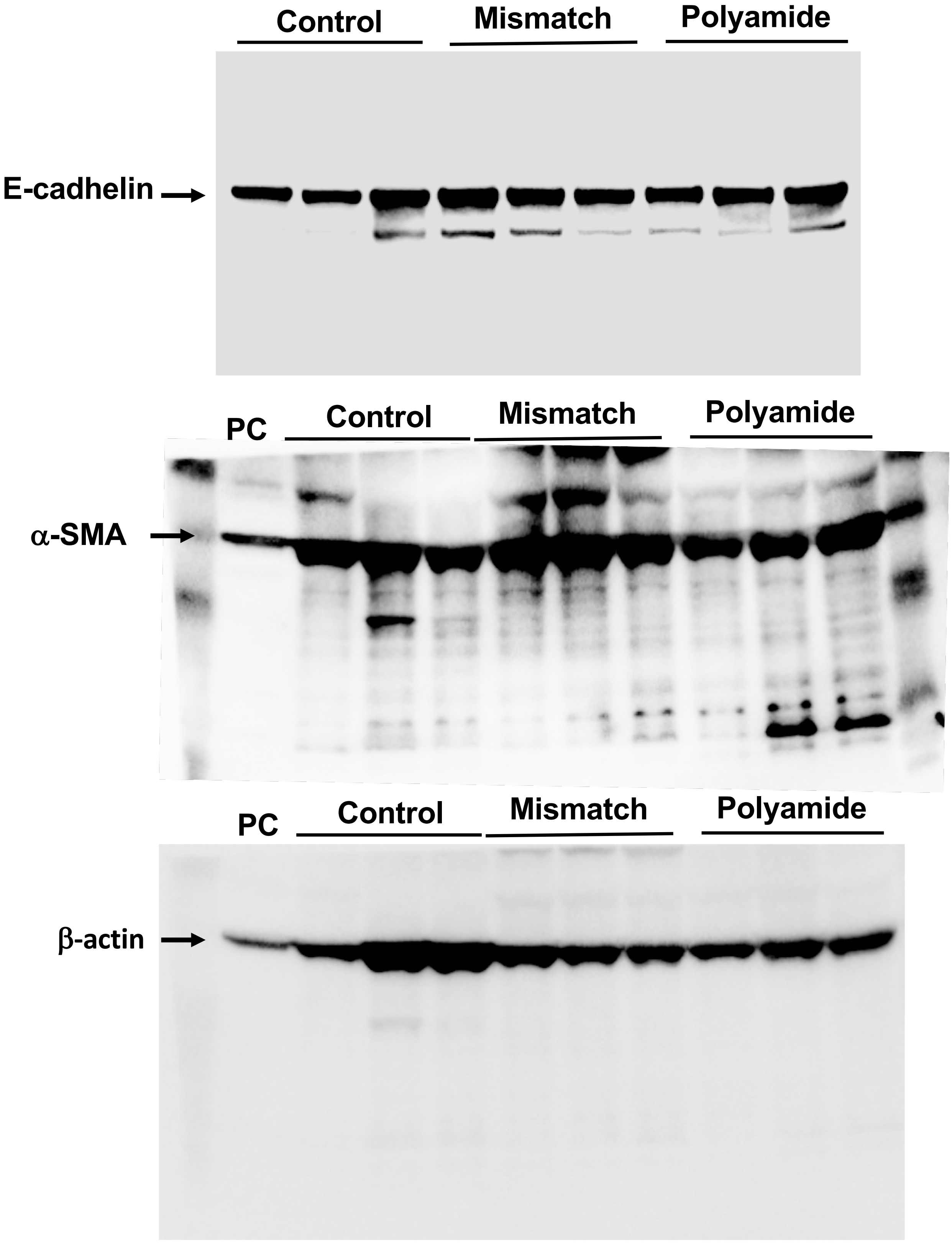

Western blot for TWIST-1 , C3, α-SMA and β-actin in UUO kidney with or without PI polyamide and Mismatch polyamide

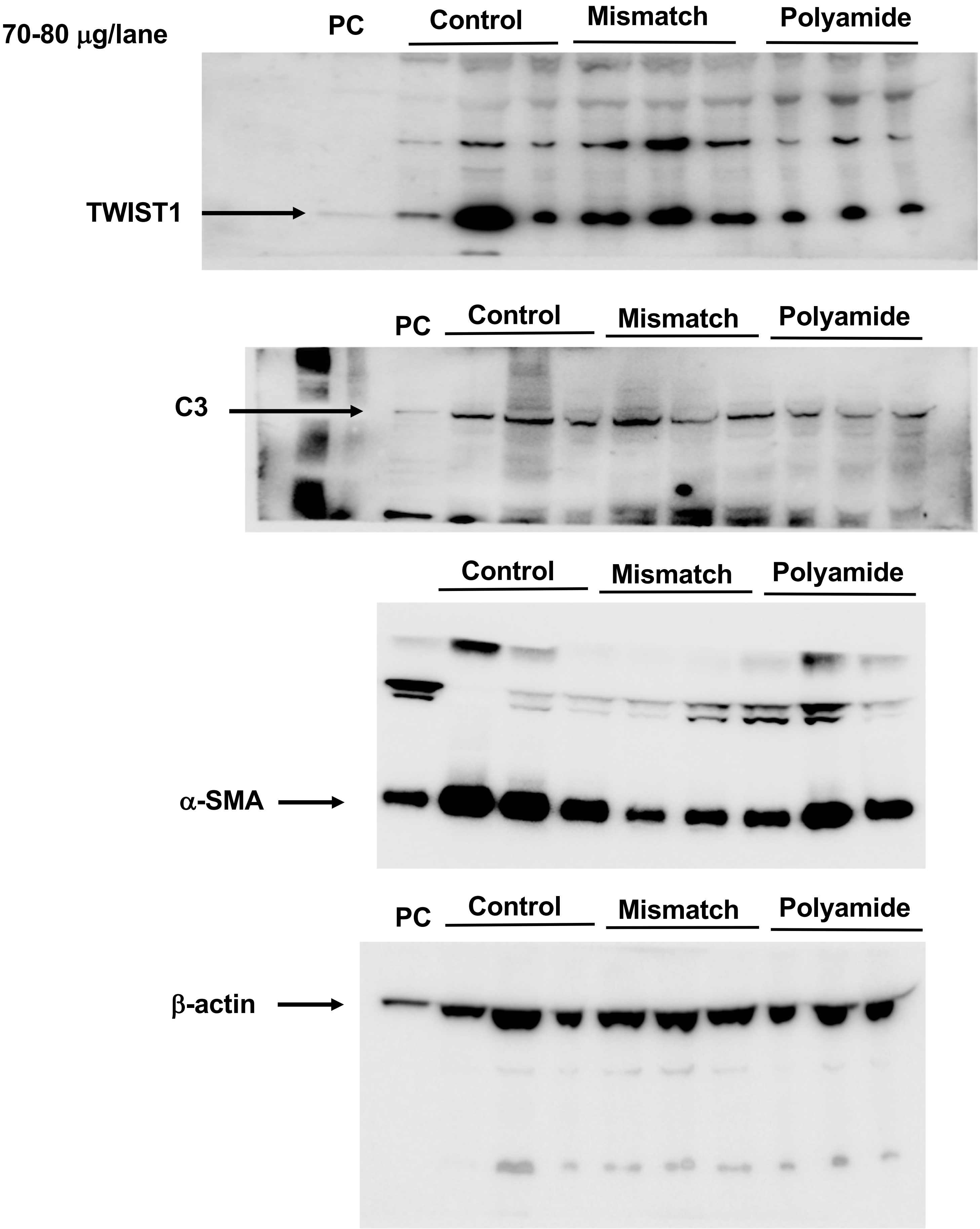

Western blot for TWIST-1 , C3, E-cadhelin, renin and β-actin in UUO kidney with or without PI polyamide and Mismatch polyamide

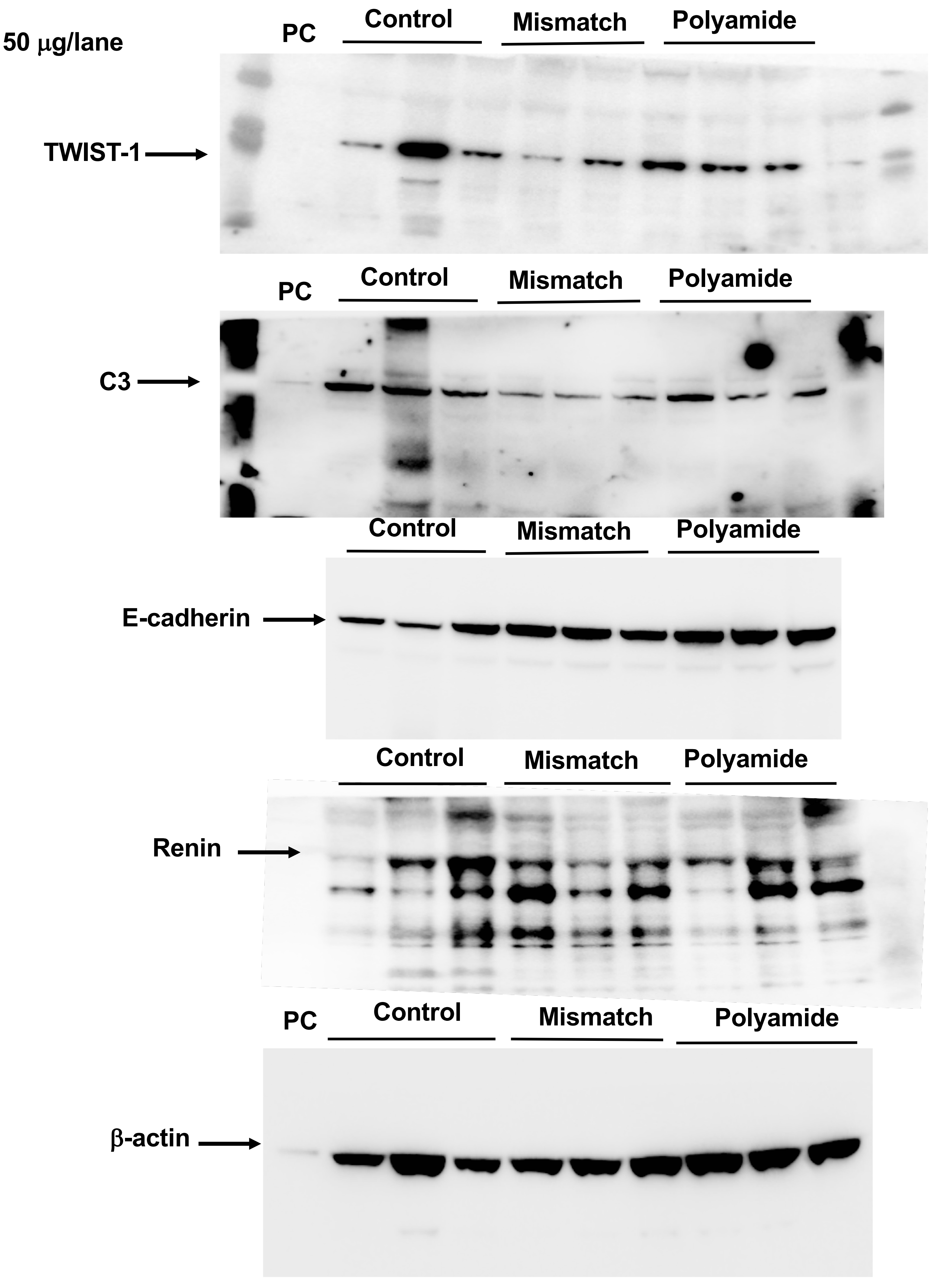

Supplement: S1 Raw images — (PDF) [file pone.0272917.s004.pdf]
